# Upgrading Common Wheat Pasta by Fiber-Rich Fraction of Potato Peel Byproduct at Different Particle Sizes: Effects on Physicochemical, Thermal, and Sensory Properties

**DOI:** 10.3390/molecules27092868

**Published:** 2022-04-30

**Authors:** Mohammad Namir, Ali Iskander, Amal Alyamani, Eman T. Abou Sayed-Ahmed, Ahmed M. Saad, Kamal Elsahy, Khaled A. El-Tarabily, Carlos Adam Conte-Junior

**Affiliations:** 1 Food Science Department, Faculty of Agriculture, Zagazig University, Zagazig 44511, Egypt; nomairmohamed@gmail.com (M.N.); iskanderali@yahoo.com (A.I.); emansayed@yahoo.com (E.T.A.S.-A.); kamalsahy@gmail.com (K.E.); 2 General Organization for Export and Import Control, Ministry of Trade and Industry, Garden City, Cairo 11519, Egypt; 3 Department of Biotechnology, Faculty of Sciences, Taif University, P.O. Box 11099, Taif 21944, Saudi Arabia; a.yamani@tu.edu.sa; 4 Biochemistry Department, Faculty of Agriculture, Zagazig University, Zagazig 44511, Egypt; 5 Department of Biology, College of Science, United Arab Emirates University, Al Ain 15551, United Arab Emirates; 6 Center for Food Analysis (NAL), Technological Development Support Laboratory (LADETEC), Federal University of Rio de Janeiro (UFRJ), Cidade Universitária, Rio de Janeiro 21941-598, Brazil; conte@iq.ufrj.br

**Keywords:** byproducts, enriched pasta, potato peel, physical properties, sensory traits

## Abstract

Fiber-enriched food has numerous health benefits. This study develops functional fiber-enriched pasta (FEP) by partially substituting wheat flour for alcohol-insoluble residue prepared from potato processing byproducts (AIR-PPB) at various particle sizes (PS). The independent variables’ effects, AIR-PPB at 2–15% substitution levels, and PS 40–250 µm were investigated in terms of chemical, cooking, thermal, and sensory properties. AIR-PPB is rich in total dietary fibers (TDF) (83%), exhibiting high water-holding capacity (WHC) and vibrant colors. Different concentrations of AIR-PPB increase TDF content in FEPs by 7–21 times compared to the control pasta (CP). Although the optimal cooking time (OCT) decreases by 15–18% compared to CP, where a lower OCT should reduce cooking time and save energy, cooking loss (Cl) increases slightly but remains within an acceptable range of 8%. Additionally, AIR-PPB altered the texture properties of FEP, with a moderate decrease in mass increase index (MII), firmness, and stickiness. AIR-PPB impairs the gluten network’s structure in pasta due to AIR-PPB’s WHC, which competes with starch for water binding, increasing the starch gelatinization temperature. FEPs show an increased lightness and yellowness and improved sensory properties. Highly acceptable FEPs were obtained for the following substitution levels: FEP11 (AIR-PPB at 2% and PS of 145 µm), FEP9 (AIR-PPB 4% level with PS of 70 µm), FEP6 (AIR-PPB of 4% level with 219 µm PS), and FEP1 (AIR-PPB = 8.5% with 40 µm PS), as compared to other FEPs.

## 1. Introduction

In recent decades, there has been a surge interest in studying the relationship between food and health. Numerous studies have examined the effects of supplementing the diet with bioactive compounds [1,2], particularly dietary fibers [3]. According to AACC [4], dietary fibers are a mixture of indigestible carbohydrates, i.e., polymers of oligo- and polysaccharides from the edible remnants of plants that resist digestion in the small intestine but are completely or partially fermented in the large intestine. Dietary fibers have been shown to have various health benefits, including improving intestinal health [5] and lowering the risk of chronic diseases such as heart disease and type 2 diabetes, high blood cholesterol, insulin resistance, obesity, and cancer [6,7]. In this regard, the recommended daily intake of dietary fibers for women is 25 g and for men is 38 g [8]. On a food technology scale, dietary fibers have a variety of functional properties that affect food quality, including water and oil holding capacity, solubility, viscosity, gel-forming ability, and swelling capacity [9]. However, dietary fibers substitution levels in food formulations are still limited due to the undesirable quality characteristics such as color, texture, and taste associated with physicochemical changes caused by fiber addition [9].

Bioactive components are abundant in industrial and agricultural wastes [10,11,12]. Furthermore, dietary fibers are derived in large part from food processing wastes. Potato (*Solanum tuberosum*) has a global production of approximately 368 million tons per year [13,14], generating a massive amount of processing wastes, equal to about 15–40% of the fresh potato and posing a potential environmental risk [15]. Most potato processing wastes consist of peels, which contain significant amounts of antioxidants and dietary fibers [16,17,18]. Additionally, several findings have shown the relationship between the quality of bakery products and the addition of potato peel fibers. The incorporation of fiber fraction into bread (0.4 g fibers/100 g flour) reduced the hardness of the bread over seven-days storage period compared to the control formulation [19]. The addition of potato peel flour with a high level of dietary fibers and protein to cakes significantly increased dietary fibers content. It decreased the cake’s hardness by 30.24%, but it had no effect on sensory traits compared to the control [20].

Pasta is typically made with an unleavened dough of wheat flour and water or eggs, formed into sheets or other shapes and cooked by boiling or baking. Pasta is broadly classified into two types: dried and fresh. Most dried pasta is commercially produced through an extrusion process [21,22]. Globally, the demand for pasta continues to grow due to its cost-effectiveness, ease of preparation, long shelf life, low glycemic index, low sodium and fat content, and high complex carbohydrates content [23,24]. According to the International Pasta Organization (IPO), global pasta production was estimated to be 14.3 million tons in 2019. As a result, pasta can serve as a vehicle for bioactive ingredients such as dietary fibers, antioxidants, omega-3 fatty acids, and protein, contributing to human health maintenance [25,26]. Numerous studies have examined the use of whole wheat flour rather than semolina to increase the nutritional value of pasta by increasing the proportion of active compounds such as dietary fibers, antioxidants, vitamins, and mineral salts found in the bran that are removed during the durum wheat milling process [26,27,28,29,30,31]. Incorporating bran as a dietary fiber source into wheat flour reduces dough development and strengthens the dough by disrupting the gluten network [32].

Additionally, the bran-enriched pasta exhibited poor cooking characteristics and a firm texture. The more fiber incorporation, the lower the sensory properties [23,33]. Thus, several trials have been conducted to enrich wheat flour with various ingredients, including oregano and carrot leaves [34] and other plant parts [35,36], white bean, split yellow pea, lentil [24], and chickpea flour, as well as protein isolates [37,38,39], quinoa flour [30], carob flour [28], and bambara groundnut flour [40]. Therefore, the challenge for food producers is to determine the most effective way to fortify pasta with dietary fibers while maintaining its quality properties.

This study aims to create functional fiber-enriched pasta (FEP) by partial substitution of wheat flour with potato peel byproducts (PPB) with different particle sizes (PS). The prepared pasta was evaluated via its proximate composition, cooking characteristics, thermal and color properties, and sensory analysis. Response surface methodology (RSM) was used to optimize the experimental processing variables for desired FEP quality attributes.

## 2. Materials and Methods

### 2.1. Materials

Potato (*Solanum tuberosum* L.) cv Spunta peel byproduct (PPB) was kindly provided by Farm Frites Factory (10th of Ramadan industrial city, Eastern Province, Egypt). Wheat flour (72% extraction rate) was purchased from a local market in Zagazig City, Zagazig, Egypt. Wheat flour contained 12.00 g/100 g, ash 0.68 g/100 g, moisture 13.41 g/100 g, total dietary fibers (TDF) 6.48 g/100 g. Following the peeling procedure, PPB samples were collected, washed with distilled water to remove impurities, and dried for 24 h in a hot air oven at 45 ± 2 °C. The dried PPB had a moisture content of 7.45 ± 0.66 g/100 g. We milled and sieved the dried samples using standard sieves. PPB fractions with PS of 40, 70, 145, 219, and 250 mm were obtained and vacuum-packed into separate plastic bags, where they were stored at 42 °C until further analysis. 

### 2.2. Preparation of Alcohol Insoluble Residue from Potato Peel Byproduct (AIR-PPB)

The AIR-PPB was prepared as described by Latorre et al. [41] with some modifications. For 30 min, 10 g of PPB samples with specified PS was stirred in 50 mL boiling ethanol (70%, *v*/*v*). The suspension was centrifuged at 3000 rpm/10 min, and the supernatant was decanted. Subsequently, the residues were collected and extracted twice for another 30 min with boiling ethanol (95%, *v*/*v*). The AIR-PPB was filtered through Whatman No.1 filter paper, washed with acetone, and the solvents were exchanged several times before being air-dried at 30 °C.

### 2.3. Experimental Design and Statistical Analysis

In order to establish an appropriate statistical model for the current study, several trials were conducted to determine the maximum and minimum amounts of AIR-PPB in pasta formulations containing various PS. Statgraphics Plus for Windows (version 4.1, Centurion, XV, USA) was used to create polynomial models. A central composite rotatable design (CCRD) was used to design the test with two independent variables (AIR-PPB 2–15 g/100 g and PS 40–250 mm) coded at five levels (−1.41, −1, 0, +1, and +1.41). To demonstrate the model’s applicability, the CCRD included 12 experiments with three replicates for the central point (Table 1). The experiments were conducted at random to mitigate the effects of unanticipated variability introduced by external sources. A second-order polynomial equation was used to investigate the relationship between the responses and the independent variables.

*Y* = b_0_ + b_1 × 1_ + b_2_X_2_ + b_11_(X_1_)^2^ + b_12_X_1_ X_2_ + b_22_(X_2_) (1)

where *Y* response value and X_1_ and X_2_ denote independent variables. *b*_0_ is a constant, *b_1_* and *b*_2_ are linear coefficients, *b*_11_ and *b*_22_ are quadratic coefficients, and *b*_12_ is a quadratic interaction coefficient.

The coefficient of determination (*R*^2^) and adjusted *R*^2^ were used to assess the fit of the model, whereas analysis of variance was used to determine the model’s significance (*p* < 0.05) (ANOVA). The correlation coefficient (*r*) was used to determine whether there was a positive (+1) or negative (−1) correlation between responses. Excel 2010 was used to calculate the means and standard deviations for the obtained results. To determine *p* < 0.05 statistically significant differences between parameters, a one-way ANOVA with Duncan’s test (*p* < 0.05) was used. The experimental processing variables were then optimized for the desired fiber-enriched pasta (FEP) quality attributes, which included a high dietary fiber content, a high sensory properties score, a shorter optimum cooking time (OCT), a lower cooking loss (Cl), and the maximum desirable color changes.

### 2.4. Preparation of FEP and Control Pasta (CP)

The experimental FEPs are primarily composed of whole wheat flour and AIR-PPB. Firstly, a Moulinex Hm4121 stand mixer (Moulinex, France) was used to combine whole wheat flour and AIR-PPB. The mixture was then hydrated by adding warm water (30 °C), which calculated to achieve a moisture content of up to 30%, and mixed at a slower speed (120 rpm) for 12 min. Finally, the mixture was fed into a pasta extruder and extruded using strand forming technology (diameter 2 mm). For 2 h, the pasta strands were dried at 80 °C. The dried FEP samples were sealed in a zip-lock plastic bag and stored at 22 ± 2 °C until further analysis. The CP was prepared (1 kg wheat, 300 mL water), except for the amount of AIR-PPB.

### 2.5. Proximate composition

Moisture, protein, fat, and ash contents were estimated as described in AOAC [42]. TDF, soluble (SDF), and insoluble (IDF) dietary fibers were determined using McCleary enzymatic and gravimetric methods, McCleary et al. [43]. Differential analysis was used to determine the solubility of carbohydrates, and each analysis was conducted in triplicates.

### 2.6. Functional Properties

The water holding capacity (WHC) was determined as described by Namir et al. [44]. In weighted test tubes, 1 g of AIR-PPB was homogenized in 10 mL of distilled water, stirred for 30 min, and then centrifuged at 6000× *g* for 30 min. The supernatant was discarded, and the residues were weighed. WHC was calculated as mL of retained water/g of sample.

### 2.7. Physical Characteristics

#### 2.7.1. Cooking Properties

OCT, min was determined in accordance with the method described by Sobota et al. [45]. The mass increase index (MII g/g) was determined by dividing the weight of cooked pasta by the weight of uncooked pasta [46]. In contrast, the Cl % was determined by measuring the solids content of the cooking water using the AACC 66-50 method [4].

#### 2.7.2. Texture Profile Analysis

TA.XT*plusC* texture analyzer (Stable Micro Systems, Godalming, UK) was used to determine the texture. Samples were cooked for the OCT and then immersed in cold water to prevent the texture from being affected by continuous cooking heat. The pasta was drained and then placed in the center of the texture analyzer platform, where it was cut at a speed of 2 mm/s and deformation of 90% with a pasta blade (thickness = 1 mm). Firmness (*N*) was defined as the maximum force required to cut a single cooked pasta, whereas stickiness (*N*) was defined as the maximum force required to separate the probe from the sample surface (*N*). For each trial, the mean of ten replicates was calculated for each trial.

#### 2.7.3. Differential Scanning Calorimeter (DSC)

The differential scanning calorimeter was used to determine the profile of starch gelatinization (Malvern Panalytical Ltd., Malvern, UK). Then, approximately 3 mg of dried flour was placed in an aluminum pan, followed by the addition of 1 mL of distilled water via micropipette in the DSC pan. The pan was sealed and left at room temperature condition for 12 h. The thermograms were taken at a rate of 5 °C/min between 25 and 120 °C. The thermogram was used to determine the starch gelatinization temperature, the onset temperature (T_o_), the peak or melting temperature (T_p_), the conclusion temperature (T_c_), and the enthalpy (*H*) values from data recording software.

### 2.8. Color Measurement and Sensory Assessment

A Color Flex EZ spectrophotometer (HunterLab, Murnau, Germany) was used to determine the color of FEP. The following parameters were taken into account: Equation (2) calculated the L*, a*, b*, and color difference (ΔE) between control pasta (CP) and FEP. The values given are the averages of three independent samples [47].

(2) $$\Delta E=\sqrt{{\left(\Delta L\right)}^{2}+{\left(\Delta a\right)}^{2}+{\left(\Delta b\right)}^{2}}$$

The sensory characteristics of FEP were evaluated using a nine-point hedonic scale (1 = strongly dislike extremely, 5 = neither like nor dislike, and 9 = like extremely). After cooking the pasta to its OCT, it was drained and rinsed. Pasta samples were randomly coded and served on white foam plates to 25 panelists, 15 males and 10 females (aged 21–30), in a fluorescent-lit laboratory with an air-conditioning temperature of 23 ± 1 °C. Water was used to rinse the panelists’ mouths between samples to avoid interfering with the results.

## 3. Results and Discussion

### 3.1. Chemical Composition and AIR-PPB Characteristics

The chemical composition, functional and color properties of AIR-PPB with various PS (40–250 µm) are listed in Table 2. AIR-PPB comprise 63.87% of the dry weight of PPB. Furthermore, TDF was the most abundant component of AIR-PPB, ranging from 82.73 to 83.02 g/100 g depending on PS, the TDF content increasing with PS increments. The TDF content of AIR-PPB was significantly higher than that of citrus peels (57 g/100 g), mango peel (51.2 g/100 g), pomegranate peel (17.53 g/100 g), and grape pomace skins (67.95 g/100 g) [48,49,50,51] but was comparable to that of passion fruit byproduct, apple, and date pomace (81.50, 82.00, and 83.70 g/100 g, respectively) [52,53]. Insoluble dietary fibers (IDF) comprise 61.39–63.10% of TDF in AIR-PPB depending on PS (40–250 µm); in contrast, soluble dietary fibers (SDF) comprise 19.92–21.34% of TDF in AIR-PPB.

The SDF content was increased in small PS due to fiber structure breakdown. Furthermore, orange byproducts, apple pomace, date pomace, and pineapple peel all demonstrated similar properties [52,54,55]. Moreover, there have been no studies conducted on the alcohol extraction of PPB. In general, fruit and vegetable byproducts are high in dietary fiber and soluble sugars but low in fat and protein [56], and the dietary fibers were combined with low protein, low fat, and soluble carbohydrates in this extraction method. Therefore, these components were identified in AIR-PPB, and their content increased as PS increased, excluding soluble carbohydrates, which exhibited the opposite trend.

The WHC of AIR-PPB was 6.73–7.75 mL/g, compared to 5.70, 4.90, and 4.12 mL/g for the date, pear, and tomato pomaces, respectively. Additionally, the WHC of the cantaloupe byproduct was 6.17 mL/g [47,57,58]. An almost identical value (7.5 mL/g) was observed in apple pomace [58]. The hydroxyl groups in polysaccharide chains may form hydrogen bonds with water, thereby increasing the water-holding capacity of fiber-rich materials [59]. The lower WHC values in AIR-PPB with smaller PS can be attributed to the degradation of polysaccharides chains, which increases the soluble fibers fraction; these chains are collapsed by centrifugation during WHC determination.

Table 2 demonstrates a significant increase in the values of lightness and yellowness in AIR-PPB with lower PS but a significant decrease in redness values. Thus, the color of the AIR-PPB changed from light brown to dark orange when PS was reduced from 250 to 40 mm, which will have a beneficial effect on the color of the FEP, as will be demonstrated later.

Industrial byproducts can differ considerably in their chemical composition. Additionally, it is important to consider that certain byproducts require special treatments and processing prior to further utilization due to their chemical composition. The chemical composition and structure of these byproducts significantly impact their functional properties and their applications. Additionally, byproducts are naturally colored differently. Therefore, high levels of these byproducts may cause color changes in the extrudates, which may or may not be desired by consumers [60].

According to ANOVA results, the moisture, protein, fat, ash, and soluble carbohydrates contents of FEP significantly affected by the substitution levels of AIR-PPB and its PS (*p* < 0.05). Additionally, the PS had a significant (*p* < 0.05) lowering effect on the FEP’s protein and fat contents. The moisture, protein, fat, ash, and soluble carbohydrate contents of all experimental FEP were significantly decreased (*p* < 0.05) compared to the CP. FEP had a slightly lower moisture content of about 4–7% compared to CP. Furthermore, FEP4 had the lowest moisture content (9.41%). Additionally, the protein content of FEP7 and FEP10 was reduced by approximately 7% compared to the CP. Although there is a slight decrease in fat content (7–17%) as compared to the CP (Table 3), FEP7, FEP10, and FEP4 had the lowest fat content (0.97, 0.98, and 0.99 g/100 g FEP, respectively) compared to CP (1.14 g/100 g CP) (Table 3). FEP4 had the lowest ash content of all FEPs (0.77 g/100 g) as compared to CP, which had an ash content of 1.28 g/100 g (Table 3). In terms of soluble carbohydrates, CP has the highest content at 85.96%, which was significantly decreased in all experimental FEP, reaching a minimum of 12% relative to control pasta in FEP4.

**Figure 1:**
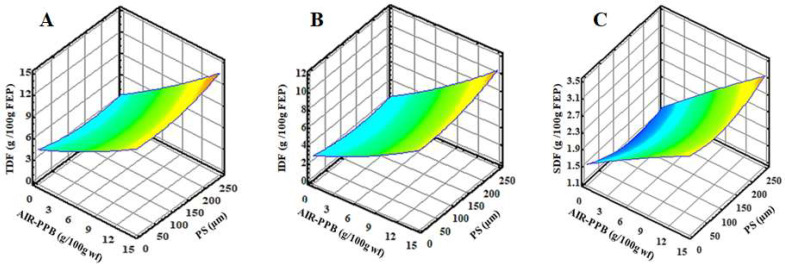
Response surface plots of the interaction of alcohol insoluble residue from potato peel byproduct (AIR-PPB) and particle size (PS) on (**A**) total dietary fibers (TDF) (g/100 g FEP), (**B**) insoluble dietary fibers (IDF) (g/100 g FEP), and (**C**) soluble dietary fibers (SDF) (g/100 g FEP) of FEP samples as compared to control pasta (CP) (0 AIR-PPB, 0 PS). FEP, fiber-enriched pasta.

The TDF content of FEP varies significantly (*p* < 0.05) between the experimental pastas. All FEPs contain more TDF than CP, which increased 7 to 21 times over CP (Table 3 and Figure 1A).

Depending on the source of the dietary fiber, fruit and vegetable byproducts may have varying amounts of insoluble and soluble fiber, which can affect their level of inclusion in direct-expanded products [56].

In comparison to CP, FEP4 had the highest TDF content of all FEPs at 12.07%, followed by FEP7, FEP10, and FEP5. According to the European Commission Regulation [61], experimental FEPs containing more than 6 g of dietary fibers per 100 g are labeled as high in dietary fibers. IDF was the predominant fraction of dietary fibers in all experimental FEP, accounting for 67–76% of TDF when compared to CP (0.38 g/100 g CP) (Table 3). The highest IDF was observed in FEP4 (9.17 g/100 g FEP), which accounted for 75.97% of TDF (Table 3, Figure 1B). The IDF is composed of cellulose, insoluble hemicelluloses, and lignin, and it plays a critical role in human and animal health, particularly in digestive processes, by promoting intestinal peristalsis, increasing fecal volume, and adsorbing heavy metals, grease, and toxic substances, which it then quickly eliminates. Additionally, SDF refers to the fraction of fibers that dissolved in water during analysis [62,63]. SDF accounts for between 24 and 33% of TDF in all experimental FEP. FEP4 had the highest SDF content (2.90 g/100 g FEP) compared to CP (0.20 g/100 g CP) (Table 3 and Figure 1C). Ritthiruangdej et al. [64] incorporated 30% unripe banana flour into the noodles, which increased the TDF and resistant starch content of noodles.

As illustrated in Table 3 and Figure 1, the second-order polynomial models for FEP characterization were significant (*p* < 0.05) and had a coefficient of determination (*R*^2^) greater than 0.90, indicating that the regression model is fit and can be used to predict the responses. Due to the unique chemical composition of AIR-PPB, the partial replacement of wheat flour with AIR-PPB at certain PS resulted in significant changes (*p* < 0.05) in all measured chemical constituents of the experimental FEP as compared to CP.

### 3.2. Physical Properties

#### 3.2.1. Cooking Properties

**Figure 2:**
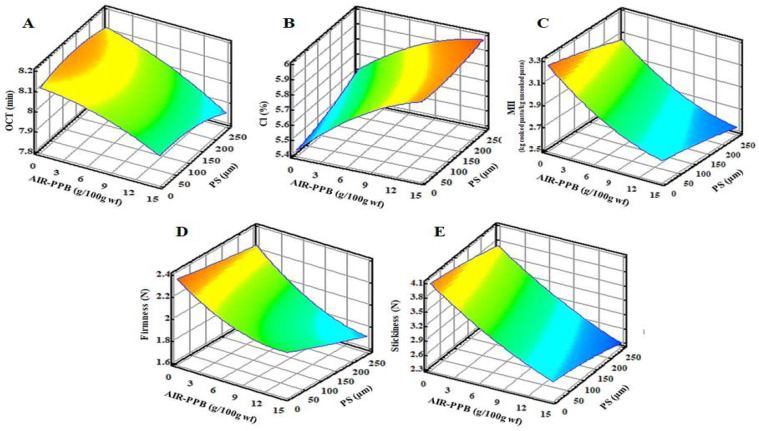
Response surface plots of the interaction of alcohol insoluble residue from potato peel byproduct (AIR-PPB) and Particle size (PS) on (**A**) optimum cooking time (OCT) (min), (**B**) cooking loss (Cl) (%), (**C**) mass increase index (MII) (kg cooked pasta/kg uncooked pasta), (**D**) Firmness (N), and (**E**) Stickiness (N) of fiber-enriched pasta (FEP) samples.

When FEPs are exposed to cooking during preparation, it is expected that heat will affect the physicochemical properties of FEP by affecting the structure of the gluten–starch network, which influences the mechanical properties of FEP. Therefore, it is critical to investigate the cooking properties of FEP in order to ascertain the effect of experimental factors on the FEP’s quality. The OCT, CL, and MII were used to evaluate the cooking properties (Table 4, Figure 2A).

Table 4 demonstrates significant (*p* < 0.05) differences in OCT values between all experimental FEPs and CP, with FEPs showing a 15–18% decrease in OCT values when compared to CP. Additionally, the higher the level of AIR-PPB substitution at high PS, the shorter the OCT (Figure 2A). The CP had the longest OCT (9.63 min), which decreased significantly by 18% and yielded the lowest FEP4 value of 7.90 min. IDF generally distribute evenly throughout the starch matrix at low AIR-PPB concentrations, strengthen the starch matrix, and may result in increased expansion. However, at higher fiber concentrations, uniform distribution is not achieved, and fiber particles may disrupt cell walls, resulting in a reduction in expansion. Additionally, IDF may compete for water with starch, preventing the starch from fully gelatinizing, increasing the melt viscosity, increasing resistance to cell formation, and decreasing cooking time, which is beneficial because a lower OCT is desired to shorten cooking time and save energy. In contrast, SDF have no adverse effect on expansion; they may result in a slight increase in pasta volume expansion [56,65]. Previous research has corroborated these findings. Pasta made from bambara groundnut enriched fractionated whole grain wheat flour had a higher PS but a lower OCT than CP made from unfractionated flour, suggesting that fibers play a role in lowering the OCT [40]. Similarly, pasta made from olive pomace fortified durum wheat semolina had an OCT of 13.30 min for the control, which decreased to 12 min for pasta fortified with 10% olive pomace [66].

Furthermore, spaghetti made from durum semolina and various proportions of durum bran had a lower OCT compared to the CP [33]. Furthermore, Lončarić et al. [67] discovered that Fettuccine enriched with 10% apple peel powder had an OCT of 7.3 min, similar to the control. However, an increase above the 10% level resulted in a decrease in OCT.

One of the most critical quality indicators in pasta is Cl%, which is defined as solids remaining in the cooking water after drying that leach from the pasta during cooking. The greater the Cl concentration, the lower the quality of the FEP obtained. As shown in Table 4, the Cl values of FEPs were significantly (*p* ˂ 0.05) higher than those of CP but within the acceptable cooking loss limit of ≤8% [68], which should not be exceeded by good-quality pasta. Increasing the AIR-PPB level with a high PS increased the Cl by 31–40% over the CP (Figure 2B). The highest Cl (5.97%) was observed in FEP4, whereas the lowest Cl (5.59%) was observed in FEP11 (4.27%). This result is explained by the interaction of AIR-PPB with the gluten–starch network, which weakens and disrupts its structure, impairing its ability to retain gelatinized starch leached from FEP into the cooking water, thereby increasing cooking loss. The addition of mango peel powder [69], carrot pomace [70], and orange dietary fiber [71] to pasta formulations revealed that at low concentrations (2.0–2.5%), these ingredients have no discernible effect on the Cl. These findings suggest that such concentrations may not significantly weaken the starch and gluten matrix. However, increasing the inclusions to 4% or 5% resulted in a significant increase in Cl in comparison to the control sample and in the treatments with a lower incorporation level.

Additionally, the results indicated a significant (*p* ˂ 0.05) reduction in MII parameter as the concentrations of AIR-PPB and PS increased (Table 4, 6, and Figure 2C). MII was highest in CP (3.51 kg cooked pasta/kg uncooked pasta) but decreased by 12–27% in all FEPs. FEP11 had the highest MII (3.08 kg CP/kg uncooked pasta with a relative decrease of 12.45%, when compared to CP, whereas FEP4 had the lowest MII (2.55 kg cp/kg up) (Table 4, Figure 2C). The observed decrease in the MII for FEP is a result of competition for water absorption between fibers’ free groups and starch, limiting the amount of available water for starch during cooking, decreasing water absorption, and consequently decreasing MII [33,72].

#### 3.2.2. Texture

The presence of the fiber-rich material may disrupt and weaken the gluten matrix within the pasta microstructure, resulting in a loss of pasta firmness. In comparison to CP (2.69 N), the experimental FEP firmness values varied significantly (*p* ˂ 0.05) from 1.68 N to 2.18 N (Table 4, Figure 2D). This revealed that increasing the level of AIR-PPB replacement with increasing PS decreased FEP firmness. Firmness was reduced to 1.68 N in FEP4 by 37.55% compared to CP (Table 4). FEP 11 and FEP 9 have the highest firmness values (2.18 N and 2.16 N, respectively) among all experimental FEPs (Table 4, Figure 2D).

In contrast, low post-cooking stickiness values indicate high-quality pasta. The 0.05 stickiness values of cooked FEP decreased significantly (*p* < 0.05) in parallel with the increase in the substitution level of AIR-PPB with high PS. The stickiness values decreased significantly (*p* < 0.05) in all FEPs compared to the CP, ranging from 22 to 49%. FEP11 had the highest stickiness (3.70 N), whereas FEP4 had the lowest (2.42 N) (Table 4, Figure 2E). The decrease in the stickiness values of FEPs can be explained by the decline in the total starch content, which is attributed to stickiness properties and is replaced by fibers in the experimental pasta. The addition of pomaces such as carrot pomace, apple peel powder, and flaxseed cake to pasta decreases its firmness/hardness [67,73]. This decreased firmness/hardness results from byproduct components that inhibit the formation of a strong gluten network [70].

The firmness of ziti-cut pasta containing 2% carrot pomace was significantly reduced from 5.94 N to 2.88 N [70]. However, increasing the number of byproducts in steps of 2% to 10% did not result in a further significant change. 

#### 3.2.3. Thermal Properties of FEP

When starch granules are heated (>60 °C) in water, they undergo a transition from an ordered to a disordered state, absorbing water, swelling, and losing crystallinity and amylopectin double-helical order in the process of gelatinization. In the present study, the thermal properties of FEP were investigated using differential scanning calorimetry (DSC), a technique that determines the gelatinization transition temperatures and enthalpy of gelatinization, both of which are affected by the molecular structure or crystallinity.

**Figure 3:**
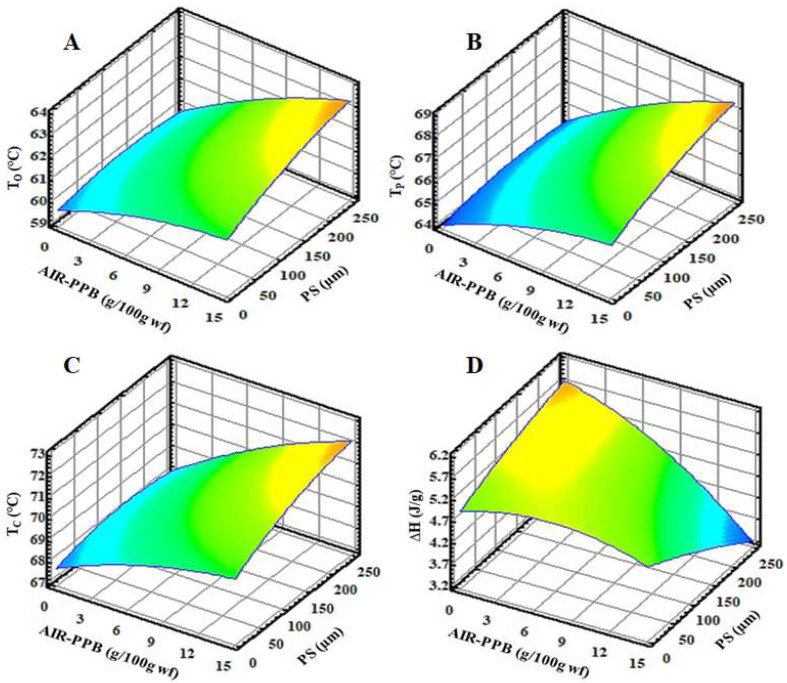
Response surface plots of the interaction of alcohol insoluble residue from potato peel byproduct (AIR-PPB) and particle size (PS) on (**A**) onset temperature (To) (°C), (**B**) melting temperature (Tp) (°C), (**C**) conclusion temperature (Tc) (°C), and (**D**) ∆H (J/g) of fiber-enriched pasta (FEP) samples.

Table 4 and Figure 3 illustrate the effect of AIR-PPB and PS on the thermal transition parameters of FEP. All experimental FEP demonstrated an endothermic transition between 60 and 71 °C, with well-defined transition temperatures (To, Tp, and Tc). These results indicated that by increasing the replacement levels of AIR-PPB and PS in FEP, the gelatinization transition temperatures (To, Tp, and Tc) increased significantly (*p* < 00.05) from 60.58 to 63.14 °C, 65.02 to 68.11 °C, and 68.83 to 71.90 °C, respectively (Table 4 and Figure 3). Because fibers and starch compete for water absorption, starch swelling and gelation events are limited, resulting in a higher starch gelatinization temperature. The gelatinization temperatures of FEP4 and FEP7 are the highest, whereas FEP9 and FEP11 are the lowest. However, they are still higher than those of CP (Table 4), implying that FEP formulations require a higher temperature to melt their crystalline structure. Similar findings have been reported for pasta enriched with pea fibers and Kañawa flour [74].

The obtained results indicated that the enthalpy value (*H*) decreased significantly (*p* < 0.05) as the amount of AIR-PPB with higher PS was increased, ranging from 3.54 to 5.49 J/g, compared to the CP (6.17 J/g) (Table 4 and Figure 3D). As reported by BeMiller [75], the loss of molecular order in the starch granule is indicated by a high enthalpy of gelatinization, which serves as a general indicator of crystallinity quantity and quality. The high WHC of AIR-PPB, particularly when combined with a high PS, inhibited swelling, lowering the starch gelatinization and enthalpy values. Similarly, pasta enriched with pea fibers, bran, and Kañawa flour demonstrated similar results [33,68,74]. Lu et al. [76] discovered that adding mushroom powder increased the *H* values of semolina-only pasta. These findings indicate that the addition of mushroom powders reduced the degree of gelatinization or dextrinization of starch granules during cold extrusion and cooking. It is speculated that mushroom fiber (particularly IDF) and fat acted as a protective agent during processing, inhibiting enzyme access to starch granules within the pasta matrix and thus limiting the release of reducing sugars during starch digestion.

### 3.3. Color Parameters and Sensory Properties

The ability of colors to attract attention has long been used by marketers to gain customers’ attention. The L*, a*, and b* values of the experimental FEPs demonstrated significant variation in the color attributes of FEP compared to CP. However, regardless of the substitution level of AIR-PPB and PS, all of the experimental FEP exhibited a tendency toward a brown color from light to dark. When AIR-PPB replacement was increased, experimental FEP became darker, more reddish, and less yellowish. Although the PS had the opposite effect on the color values, increasing lightness, redness, and yellowness values as the PS was decreased. This behavior is explicable by examining the color of AIR-PPB at various PS. The findings corroborated those of Makhlouf et al. [77] who incorporated oat bran (OB), whole barley flour, and resistant starch (RS) into pasta formulations at various concentrations. They concluded that among the three fiber sources, only OB concentrations caused the apparent changes to the lightness (L*) and red-to-green color coordinate (a*). All three fibers’ source materials altered the b* value (blue-to-yellow coordinate), resulting in all samples having a more yellowish hue compared to the control product.

The differences in color (∆E) results confirmed the effect of AIR-PPB replacement levels and PS on the color values of FEP. The ∆E values of FEP were significantly different (*p* ˂ 0.05) and showed an increasing tendency as the AIR-PPB replacement level and its PS increased (Table 5). All experimental FEP demonstrated significant and observable differences in color (*p <* 0.05) according to the color difference findings. FEP4 had the highest value of color change (19.66); however, FEP 11 had the lowest value of color change (8.40) (Table 5).

Sensory evaluation is a critical indicator for determining the optimal conditions for FEP production because it is related to the product’s acceptability and marketability on a commercial scale. It is possible to determine the acceptable ranges of AIR-PPB addition and its PS, as well as the expected physicochemical properties, based on the sensory evaluation.

Table 5 compares the sensory properties of experimental FEP to those of CP. Enriching pasta with AIR-PPB at the experimental PS can result in a significant (*p* < 0.05) reduction in color, taste, flavor, texture, and overall acceptability, which could be attributed to the interaction of the macromolecules of wheat dough with AIR-PPB at the experimental PS, resulting in variation in FEP qualities. Although the CP attributes had the highest preference scores (8.76 for color, 8.91 for taste, 8.09 for flavor, 8.93 for texture, and 8.87 for overall acceptability), the values obtained from FEP1, 6, 9, and 11 were greater than 7 for all sensory attributes compared to the CP. Meanwhile, sensory scores for FEP-2, 3, 8 (center point), 4, 5, 7, and 10 were the lowest (Table 5). The vibrant yellow color of pasta is a critical factor in determining consumer acceptance. Carotenoid pigments, in particular, are responsible for the yellow color of pasta. Semolina’s ash content also contributes to its coloring. Increased ash content results in the pale color of pasta. Sobotaet al. [78] showed that consumers prefer darker pasta because they think it is healthier because it contains fiber. Makhlouf et al. [77] demonstrated that FEP could be produced by adding up to 15% of dietary fiber into regular semolina-based pasta formulation, leading to acceptable products with matching characteristics of texture and color compared to commercial products. Among the three fiber sources, OB offered better characteristics of texture and taste, whereas RS featured the most desired golden color. Although all three addition levels resulted in acceptable products, the lower addition level (5 per cent) led to the highest preference from the sensory panel.

Table 6 shows that the color, taste, and flavor scores of FEP were negatively correlated with the TDF content (*r* = −0.91, −0.89, and −0.88) and the Cl content (−0.95, −0.94, and −0.94), respectively, confirming the hypothesis of dietary fibers having a negative effect on sensory properties, as well as the negative impact of micro-molecules of FEP leaching into the cooking water as a result of the gluten network’s weakness. Notably, the greater the color change, the less acceptable the sensorial color (*r* = −0.76). In addition, there was a negative correlation between texture score and TDF content (r = −0.87). Conversely, we observed a positive correlation between firmness and stickiness (r = 0.86). The interaction of AIR-PPB with starch results in a decrease in the amount of gelatinized starch required to maintain the structural characteristics of dough and disrupts the starch–protein matrix [79]. Similarly, ANOVA and RSM results indicated that overall acceptability scores were negatively correlated with TDF (r = −0.89), whereas ∆E (*r* = −0.73) was negatively correlated with increasing FEP firmness and stickiness (*r* = 0.88).

From the previous results and RSM results, the highly acceptable FEP samples were in descending order: FEP11 (AIR-PPB = 2 g/100 g wheat flour, PS = 145 µm), FEP9 (AIR-PPB = 4 g/100 g wheat flour, PS = 70 µm), FEP6 (AIR-PPB = 4 g/100 g wheat flour, PS = 219 µm), and FEP1 (AIR-PPB = 8.50 g/100 g wheat flour, PS = 40 µm); TDF concentrations were 4.12, 5.55, 5.64, and 7.80 g/100 g FEP in these fortified samples. Daily consumption of the recommended 25 g of dietary fibers requires 606.80, 450.45, 443.26, and 320.51 g of FEP11, FEP9, FEP6, and FEP1, respectively, in comparison to the CP (4310.34 g). Notably, the OCT values at these points were 8.14, 8.09, 8.07, and 8.05 min, respectively, compared to CP. Additionally, Cl values were 5.59, 5.65, 5.73, and 5.76% higher than CP values. Firmness values were 2.18, 2.16, 2.01, and 1.98 N, respectively, whereas stickiness values were 3.70, 3.58, 3.24, and 2.97 N, corresponding to CP. The values for color changes were 8.40, 8.62, 9.32, and 9.87. The temperatures used for gelation were 65.02, 65.42, 65.71, and 65.79 ºC, respectively.

## 4. Conclusions

Fiber supplementation improves digestion and treats a variety of recent malnutrition-related diseases. In this study, we developed a cost-effective FEP pasta by incorporating AIR-PPB into whole wheat flour. The fiber-rich fraction (AIR-PPB) was successfully isolated from PPB and used as a promising source of fibers in FEB at a variety of substitution levels and PS. The TDF, WHC, and significant color characteristics, particularly (L*, b*), distinguish the AIR-PPB with different PS. These unique properties of AIR-PPB correlate positively with the color and sensory quality of pasta. Additionally, there is a positive effect on the gluten network and thermal properties of starch gelatinization, resulting in improved cooking properties and texture compared to CP. The optimal processing parameters obtained via the sensory evaluation revealed that FEP 11, 9, 6, and 1 have the highest acceptability. Additionally, these formulations can help individuals meet the recommended daily intake of dietary fibers. Therefore, this study recommends using AIR-PPB at specific PS as a suitable dietary fiber source for commercial FEP. 

## Figures and Tables

**Table 1:** Experimental design values for fiber-enriched pasta (FEP) with different combinations of alcohol insoluble residue from potato peel byproduct (AIR-PPB) (X_1_) and particle size PS (X_2_).

Experiments	Independent Variables	
	Coded/Real Values	
	X_1_, AIR-PPB (g/100 g Wheat Flour)	X_2_, PS (µm)
FEP-1	0 (8.50)	−1.41 (40)
FEP-2 (Central point)	0 (8.50)	0 (145)
FEP-3 (Central point)	0 (8.50)	0 (145)
FEP-4	+1 (13)	+1 (219)
FEP-5	0 (8.50)	+1.41 (250)
FEP-6	−1 (4)	+1 (219)
FEP-7	+1.41 (15)	0 (145)
FEP-8 (Central point)	0 (8.50)	0 (145)
FEP-9	−1 (4)	−1 (70)
FEP-10	+1 (13)	−1 (70)
FEP-11	−1.41 (2)	0 (145)
Control pasta (CP)	0	-

**Table 2:** Proximate composition, water holding capacity (WHC), and color attributes of alcohol insoluble residue from potato peel byproduct (AIR-PPB) at different particle sizes (PS).

Composition^•^(g/100 g)	Particles’ Size (µm)				
	250	219	145	70	40
**Chemical**					
Moisture (w.b) *	5.78 ± 0.03 ^a^	5.67 ± 0.06 ^b^	5.61 ± 0.00 ^bc^	5.62 ± 0.04 ^bc^	5.55 ± 0.01 ^c^
Protein	5.46 ± 0.13 ^a^	5.40 ± 0.18 ^a^	5.38 ± 0.09 ^ab^	5.26 ± 0.04 ^c^	5.29 ± 0.21 ^bc^
Fat	0.54 ± 0.01 ^a^	0.50 ± 0.00 ^ab^	0.51 ± 0.00 ^ab^	0.45 ± 0.01 ^ab^	0.42 ± 0.00 ^b^
Ash	4.71 ± 0.23 ^a^	4.69 ± 0.09 ^ab^	4.61 ± 0.13 ^bc^	4.64 ± 0.17 ^ab^	4.53 ± 0.26 ^c^
TDF••	83.02 ± 0.45 ^a^	82.91 ± 0.31 ^b^	82.87 ± 0.17 ^c^	82.81 ± 0.25 ^cd^	82.73 ± 0.21 ^d^
IDF	63.10 ± 0.51 ^a^	62.54 ± 0.35 ^b^	62.15 ± 0.43 ^c^	61.86 ± 0.41 ^d^	61.39 ± 0.21 ^e^
SDF	19.92 ± 0.11 ^e^	20.37 ± 0.10 ^d^	20.72 ± 0.09 ^c^	20.95 ± 0.13 ^b^	21.34 ± 0.24 ^a^
SC•••	6.27 ± 0.31 ^d^	6.50 ± 0.26 ^d^	6.63 ± 0.41 ^c^	6.84 ± 0.09 ^b^	7.03 ± 0.81 ^a^
**Physical**					
WHC (mL/g)	7.75 ± 0.19 ^a^	7.61 ± 0.88 ^b^	7.21 ± 0.34 ^c^	7.04 ± 0.56 ^d^	6.73 ± 0.61 ^e^
L*	64.22 ± 0.83 ^e^	66.14 ± 0.00 ^d^	69.79 ± 0.63 ^c^	74.34 ± 0.97 ^b^	75.12 ± 0.53 ^a^
a*	15.52 ± 0.68 ^a^	11.65 ± 0.56 ^b^	9.65 ± 0.00 ^c^	8.32 ± 0.66 ^d^	7.24 ± 0.74 ^e^
b*	48.26 ± 0.28 ^e^	51.76 ± 0.00 ^d^	55.45 ± 0.21 ^c^	66.31 ± 0.20 ^b^	68.81 ± 0.37 ^a^

*•: wet basis, •• TDF: total dietary fibers, IDF: insoluble dietary fibers, SDF: soluble dietary fibers, ••• SC: soluble carbohydrates. Values are expressed as the mean ± SD of three replicates. Means within a row with different superscript letters are significantly different at the level of *p* < 0.05. L*: whiteness, a*: redness, b*: yellowness.

**Table 3:** Chemical composition of fiber-enriched pasta (FEP) (g/100 g).

Chemical Composition	CP	FEP-1	FEP-2,3,8	FEP-4	FEP-5	FEP-6	FEP-7	FEP-9	FEP-10	FEP-11
Moisture	10.08 ± 0.12 ^a^	9.67 ± 0.00 ^c^	9.59 ± 0.11 ^f^	9.41 ± 0.32 ^j^	9.54 ± 0.27 ^g^	9.62 ± 0.78 ^e^	9.45 ± 0.14 ^i^	9.65 ± 0.00 ^d^	9.49 ± 0.01 ^h^	9.72 ± 0.02 ^b^
Protein	11.04 ± 0.27 ^a^	10.48 ± 0.16 ^g^	10.56 ± 0.13 ^f^	10.32 ± 0.09 ^h^	10.59 ± 0.12 ^e^	10.81 ± 0.40 ^c^	10.23 ± 0.10 ^i^	10.79 ± 0.17 ^d^	10.25 ± 0.22 ^i^	10.89 ± 0.30 ^b^
Fat	1.14 ± 0.10 ^a^	1.01 ± 0.01 ^d^	1.02 ± 0.04 ^d^	0.99 ± 0.07 ^e^	1.04 ± 0.03 ^c^	1.05 ± 0.02 ^c^	0.97 ± 0.00 ^e^	1.04 ± 0.02 ^c^	0.98 ± 0.00 ^e^	1.07 ± 0.06 ^b^
TDF	0.58 ± 0.09 ^j^	7.80 ± 0.15 ^f^	8.08 ± 0.19 ^e^	12.07 ± 0.08 ^a^	9.45 ± 0.12 ^d^	5.64 ± 0.26 ^g^	11.88 ± 0.32 ^b^	5.55 ± 0.38 ^h^	10.41 ± 0.29 ^c^	4.12 ± 0.21 ^i^
IDF	0.38 ± 0.02 ^j^	5.54 ± 0.07 ^f^	5.90 ± 0.13 ^e^	9.17 ± 0.21 ^a^	6.99 ± 0.16 ^d^	3.95 ± 0.10 ^g^	9.03 ± 0.14 ^b^	3.83 ± 0.25 ^h^	7.81 ± 0.27 ^c^	2.76 ± 0.17 ^i^
SDF	0.20 ± 0.00 ^j^	2.26 ± 0.01 ^e^	2.18 ± 0.00 ^f^	2.90 ± 0.06 ^a^	2.46 ± 0.01 ^d^	1.69 ± 0.00 ^h^	2.85 ± 0.00 ^b^	1.72 ± 0.11 ^g^	2.60 ± 0.00 ^c^	1.36 ± 0.05 ^i^
Ash	1.28 ± 0.13 ^a^	1.09 ± 0.12 ^e^	1.00 ± 0.17 ^f^	0.77 ± 0.00 ^j^	0.89 ± 0.00 ^g^	1.13 ± 0.02 ^d^	0.84 ± 0.00 ^h^	1.19 ± 0.20 ^c^	0.82 ± 0.00 ^i^	1.22 ± 0.01 ^b^
SC	85.96 ± 1.54 ^a^	79.62 ± 0.81 ^e^	79.33 ± 1.91 ^f^	75.86 ± 1.45 ^j^	78.03 ± 1.51 ^g^	81.37 ± 1.20 ^d^	76.07 ± 0.92 ^i^	81.43 ± 0.81 ^c^	77.53 ± 1.53 ^h^	82.69 ± 0.40 ^b^

Values are means ± SD. Means within a row with different superscript letters are significantly different (*p* < 0.05). FEP-2,3,8 is the center point, and their value is the average of the three central points. TDF: total dietary fibers; IDF: insoluble dietary fibers; SDF: soluble dietary fibers; SC: soluble carbohydrates, CP: control pasta.

**Table 4:** Physical properties (cooking, texture, thermal) of fiber-enriched pasta (FEP).

Physical Properties	CP	FEP-1	FEP-2,3,8	FEP-4	FEP-5	FEP-6	FEP-7	FEP-9	FEP-10	FEP-11
**Cooking**										
OCT (min)	9.63 ± 1.21 ^a^	8.05 ± 1.45 ^e^	8.04 ± 0.64 ^e^	7.90 ± 0.72 ^i^	7.99 ± 0.56 ^f^	8.07 ± 0.78 ^d^	7.93 ± 0.68 ^h^	8.09 ± 0.42 ^c^	7.96 ± 0.81 ^g^	8.14 ± 0.46 ^b^
Cl (%)	4.27 ± 0.16 ^i^	5.76 ± 0.10 ^e^	5.83 ± 0.15 ^d^	5.97 ± 0.06 ^a^	5.88 ± 0.24 ^c^	5.73 ± 0.77 ^f^	5.92 ± 0.80 ^b^	5.65 ± 0.81 ^g^	5.91 ± 0.31 ^b^	5.59 ± 0.88 ^h^
MII (kgcp/kg up)	3.51 ± 0.86 ^a^	2.79 ± 0.73 ^e^	2.74 ± 0.89 ^f^	2.55 ± 0.31 ^i^	2.72 ± 0.10 ^g^	2.82 ± 0.21 ^d^	2.60 ± 0.00 ^h^	2.98 ± 0.17 ^c^	2.71 ± 0.30 ^g^	3.08 ± 0.46 ^b^
**Texture**										
Firmness (N)	2.69 ± 0.95 ^a^	1.98 ± 0.36 ^d^	1.91 ± 0.44 ^f^	1.68 ± 0.24 ^h^	1.92 ± 0.34 ^ef^	2.01 ± 0.10 ^c^	1.85 ± 0.21 ^g^	2.16 ± 0.21 ^b^	1.93 ± 0.67 ^e^	2.18 ± 0.58 ^b^
Stickiness (N)	4.73 ± 0.31 ^a^	2.97 ± 0.28 ^e^	2.95 ± 0.26 ^f^	2.42 ± 0.30 ^j^	2.88 ± 0.00 ^g^	3.24 ± 0.93 ^d^	2.45 ± 0.87 ^i^	3.58 ± 0.80 ^c^	2.83 ± 0.16 ^h^	3.70 ± 0.01 ^b^
**Thermal properties**										
To (°C)	59.44 ± 1.38 ^j^	61.16 ± 1.09 ^f^	61.99 ± 1.27 ^e^	63.14 ± 1.52 ^a^	62.14 ± 1.47 ^d^	60.88 ± 1.22 ^g^	62.54 ± 1.43 ^b^	60.72 ± 1.29 ^h^	62.33 ± 1.34 ^c^	60.58 ± 1.71 ^i^
Tp (°C)	63.97 ± 1.33 ^j^	65.79 ± 1.61 ^f^	66.78 ± 1.39 ^e^	68.11 ± 1.23 ^a^	66.96 ± 1.13 ^d^	65.71 ± 1.82 ^g^	67.47 ± 1.51 ^b^	65.42 ± 1.57 ^h^	67.18 ± 1.54 ^c^	65.02 ± 1.69 ^i^
Tc (°C)	66.54 ± 1.42 ^j^	69.52 ± 1.37 ^f^	70.58 ± 1.61 ^e^	71.90 ± 1.82 ^a^	70.72 ± 1.83 ^d^	69.44 ± 1.76 ^g^	71.26 ± 1.22 ^b^	69.18 ± 1.31 ^h^	70.96 ± 1.49 ^c^	68.83 ± 1.71 ^i^
*H* (J/g)	6.17 ± 0.98 ^a^	5.19 ± 0.91 ^d^	4.89 ± 0.43 ^e^	3.54 ± 0.61 ^i^	4.87 ± 0.28 ^f^	5.21 ± 0.19 ^d^	4.13 ± 0.75 ^h^	5.28 ± 0.21 ^c^	4.64 ± 0.19 ^g^	5.49 ± 0.85 ^b^

* Values are means ± SD. Means within a row with different superscript letters are significantly different (*p* < 0.05). FEP-2,3,8 is the center point, and its value is the average of the three central points. CP: control pasta, OCT: optimum cooking time (min), Cl: cooking loss (%), MII: mass increase index (kg cooked pasta/kg uncooked pasta), To: onset temperature (°C), Tp: melting temperature (°C), Tc: conclusion temperature (°C) and *H*: enthalpy value.

**Table 5:** Color parameters and sensorial traits of fiber-enriched pasta (FEP).

Color Parameters	CP	FEP-1	FEP-2,3,8	FEP-4	FEP-5	FEP-6	FEP-7	FEP-9	FEP-10	FEP-11
L*	68.82 ± 0.00 ^a^	59.02 ± 0.02 ^e^	58.66 ± 0.00 ^f^	49.21 ± 0.02 ^j^	57.14 ± 0.00 ^g^	59.52 ± 0.01 ^d^	49.56 ± 0.01 ^i^	60.22 ± 0.01 ^c^	54.32 ± 0.06 ^h^	60.43 ± 0.00 ^b^
a*	0.84 ± 0.04 ^i^	1.79 ± 0.03 ^a^	1.61 ± 0.07 ^c^	1.32 ± 0.03 ^f^	1.51 ± 0.06 ^d^	1.19 ± 0.08 ^h^	1.38 ± 0.02 ^e^	1.27 ± 0.01 ^g^	1.64 ± 0.09 ^b^	1.19 ± 0.01 ^h^
b*	13.24 ± 0.01 ^a^	12.46 ± 0.08 ^e^	12.26 ± 0.03 ^f^	11.80 ± 0.00 ^j^	12.24 ± 0.00 ^g^	12.60 ± 0.00 ^d^	11.93 ± 0.05 ^i^	12.71 ± 0.00 ^c^	11.97 ± 0.01 ^h^	12.78 ± 0.04 ^b^
ΔE	----	9.87 ± 0.03 ^f^	10.23 ± 0.01 ^e^	19.66 ± 0.03 ^a^	11.74 ± 0.01 ^d^	9.32 ± 0.03 ^g^	19.31 ± 0.02 ^b^	8.62 ± 0.01 ^h^	14.57 ± 0.04 ^c^	8.40 ± 0.03 ^i^
**Sensory properties •**										
Color	8.76 ± 1.96 ^a^	8.11 ± 1.70 ^e^	6.71 ± 1.25 ^f^	5.86 ± 1.36 ^j^	6.54 ± 1.22 ^g^	8.32 ± 1.20 ^d^	6.19 ± 1.10 ^i^	8.56 ± 1.82 ^c^	6.46 ± 1.70 ^h^	8.61 ± 1.09 ^b^
Taste	8.91 ± 1.84 ^a^	7.94 ± 1.68 ^e^	6.54 ± 1.19 ^f^	5.93 ± 1.83 ^j^	6.40 ± 1.37 ^g^	8.09 ± 1.27 ^d^	6.13 ± 0.98 ^i^	8.37 ± 1.18 ^c^	6.29 ± 1.67 ^h^	8.41 ± 1.10 ^b^
Flavor	8.09 ± 1.57 ^a^	7.44 ± 1.64 ^e^	6.03 ± 1.27 ^f^	5.58 ± 1.56 ^j^	5.91 ± 1.61 ^g^	7.54 ± 1.02 ^d^	5.69 ± 1.12 ^i^	7.85 ± 1.77 ^c^	5.78 ± 1.61 ^h^	7.88 ± 1.45 ^b^
Texture	8.93 ± 1.40 ^a^	7.83 ± 1.82 ^e^	6.43 ± 1.34 ^f^	6.02 ± 1.79 ^j^	6.33 ± 1.52 ^g^	7.92 ± 1.17 ^d^	6.13 ± 1.17 ^i^	8.17 ± 1.01 ^c^	6.22 ± 1.89 ^h^	8.21 ± 1.27 ^b^
Overall acceptability	8.87 ± 1.21 ^a^	8.07 ± 1.93 ^e^	6.67 ± 1.31 ^f^	6.05 ± 1.83 ^j^	6.53 ± 1.41 ^g^	8.22 ± 1.08 ^d^	6.26 ± 1.09 ^i^	8.49 ± 1.89 ^c^	6.43 ± 1.32 ^h^	8.53 ± 1.13 ^b^

* Values are means ± SD. Means within a row with different superscript letters are significantly different (*p* < 0.05). • Mean values of hedonic test from 25 panelists (15 males and 10 females at 21–30 years old), FEP-2,3,8 is the center point, and its value is the average of the three central points. CP: control pasta, L*: whiteness, a*: redness, b*: yellowness’, ΔE: color difference.

**Table 6:** Data analysis of the effect of alcohol insoluble residue from potato peel byproduct (AIR-PPB) and particle size (PS) on chemical and physical properties of fiber-enriched pasta (FEP).

	TDF (g/100 g)		OCT (min)		Cl (%)		MII (Kg CP/kg Up)		Firmness (N)		Stickiness (N)		∆E		To (°C)		Tp (°C)		Tc (°C)		*H* (J/g)	
Source	*F.*	*p.*	*F.*	*p.*	*F.*	*p.*	*F.*	*p.*	*F.*	*p.*	*F.*	*p.*	*F.*	*p.*	*F.*	*p.*	*F.*	*p.*	*F.*	*p.*	*F.*	*p.*
X_1_	1760.54	0.0000	275.23	0.0000	336.51	0.0000	88.88	0.0002	40.92	0.0014	141.97	0.0001	328.18	0.0000	106.53	0.0001	118.31	0.0001	108.85	0.0001	54.34	0.0007
X_2_	45.51	0.0011	20.04	0.0065	39.15	0.0015	11.07	0.0209	10.20	0.0242	10.77	0.0219	18.90	0.0074	13.75	0.0139	16.86	0.0093	15.85	0.0105	7.24	0.0433
X_1 × 1_	2.96	0.1460	4.78	0.0804	10.83	0.0217	3.11	0.1383	2.76	0.1573	1.15	0.3334	80.56	0.0003	1.78	0.2401	1.74	0.2438	1.68	0.2511	2.94	0.1473
X_1 × 2_	9.04	0.0299	1.82	0.2355	1.72	0.2461	0.09	0.7756	0.32	0.5945	0.00	0.9823	8.52	0.0330	2.45	0.1781	1.95	0.2217	1.98	0.2180	5.85	0.0602
X_2 × 2_	16.32	0.0099	5.11	0.0734	1.22	0.3189	0.06	0.8107	0.01	0.9302	0.13	0.7344	5.95	0.0587	1.36	0.2965	0.99	0.3644	1.61	0.2599	0.12	0.7424
*R* ^2^	99.74		98.5367		98.8226		95.6893		92.2817		97.0473		98.9435		96.3575		96.7292		96.452		94.1099	
*R*^2^ (d.f.)	99.4955		97.0734		97.6453		91.3785		84.5634		94.0945		97.887		92.715		93.4583		92.904		88.2198	
MAE	0.0946195		0.0071620		0.0095813		0.0227303		0.0275063		0.0521069		0.283463		0.113512		0.121282		0.122567		0.0977168	

MAE: Mean absolute error, F: *F*-Ratio, *p*: *p*-Value, TDF: total dietary fibers, OCT: optimum cooking time (min), Cl: cooking loss (%), MII: mass increase index (kg cooked pasta/kg uncooked pasta), ∆E: color difference, To: onset temperature, Tp: melting temperature, Tc: conclusion temperature, *H*: enthalpy value.

## Data Availability

Not applicable.

## Sample Availability

Samples of the compounds are not available from the authors.
